# Skeletal myopathy in Pompe disease: a failure of satellite cell activation?

**DOI:** 10.1186/s40478-018-0638-6

**Published:** 2018-11-30

**Authors:** Annie Hiniker, Marta Margeta

**Affiliations:** 1 Department of Pathology, University of California, San Diego, San Diego, CA USA; 2 Department of Pathology, University of California, San Francisco, San Francisco, CA USA

**Keywords:** Pompe disease, Acid maltase, Satellite cells, Regeneration, Autophagy

Pompe disease, also known as glycogen storage disease type II or acid maltase deficiency, is a lysosomal storage disorder caused by loss-of-function mutations in acid α-glucosidase (GAA), the sole lysosomal enzyme that catabolizes glycogen to glucose [4]. Some GAA mutations abolish all enzymatic activity and cause severe infantile Pompe disease with cardiac muscle involvement and death in the first year of life; other mutations allow residual GAA activity, and patients live to adulthood with skeletal muscle weakness as the main source of morbidity and mortality [14]. While enzyme replacement therapy (ERT; approved for treatment of Pompe disease in 2006) robustly improves cardiac function, it has little effect on skeletal muscle [7, 8]; a better understanding of the mechanism that underlies progressive skeletal muscle dysfunction in Pompe disease is therefore critical for further therapeutic advancements.

Two papers recently published in Acta Neuropathologica Communications (one by Colle group in France (Lagalice et al. [2]) and the other by Pijnappel group in Netherlands (Schaaf et al. [11]) provide an interesting insight into pathogenesis of Pompe skeletal myopathy by presenting  compelling evidence that satellite cell activation may be a key step at which skeletal muscle repair fails in Pompe disease. Investigators in both groups used murine models of Pompe disease (different strains of acid alpha-glucosidase knockout mice; Gaa−/−) to perform longitudinal studies of the skeletal muscle pathology and to investigate the functionality of the muscle regenerative response; the results of these two studies, which are robust and on the whole beautifully reproduce each other, build on the previous findings obtained using Pompe mouse models and skeletal muscle tissue from the Pompe disease patients. Both papers show that the Gaa−/− mice develop abundant skeletal muscle glycogen accumulation that can be detected as early as 2 weeks of age but plateaus soon thereafter (by 1.5 months or 25 weeks, depending on the time point examined in each study). This is followed by progressive failure of autophagy (age-dependent accumulation of LC3-positive aggregates and enlarged lysosomes) that is documented in particular detail in the paper by Lagalice et al. Both groups also demonstrate a progressive increase in the central nucleation while failing to detect a significant population of actively regenerating fibers (measured by immunohistochemical detection of embryonic myosin heavy chain [eMyHC] protein). In addition, both groups document an age-dependent decrease in the average muscle fiber diameter accompanied by a loss of large fibers, with paper by Lagalice et al. also demonstrating an age-dependent increase in the number of split fibers. Taken together, these findings point to an impairment of muscle regenerative response, which requires satellite cell activation [3, 5, 9].

Satellite cells are Pax7-positive multipotent cells that in a quiescent state lie between the sarcolemma and the basement membrane. In response to muscle fiber injury, they become activated and proliferate, with some progeny undergoing myogenic differentiation and expression of eMyHC, while other progeny replenishes the satellite cell reserve. Recent work on human samples (previously also published in ANC by the Pijnappel group [10]) demonstrated that the Pax7-positive satellite cell pool appears to be completely preserved in Pompe patients, providing indirect support for a model in which impaired satellite cell activation contributes to Pompe disease pathogenesis. Lagalice et al. and Schaaf et al. therefore used the Gaa−/− mice to directly interrogate muscle regenerative capacity and the function of satellite cells in the Pompe model system. Excitingly, both groups found the satellite cell pool in the Gaa−/− mice to be preserved *and* fully functional in response to acute muscle injury: After myotoxicity was induced by treatment with either cardiotoxin or barium chloride, Gaa−/− mice showed efficient muscle regeneration that was essentially indistinguishable from that of WT mice. Additionally, Schaaf et al. performed multiple rounds of injury and recovery, and found that even after 3 consecutive injuries to the same muscle, Gaa−/− mice showed complete regeneration as well as return of satellite cells to quiescence.

What does this mean for the Pompe disease treatment? Based on the findings from these two studies, skeletal muscle in Pompe disease shows preserved regenerative potential and responds appropriately to acute injury, but does not robustly regenerate in response to pathogenic glycogen accumulation. Thus, satellite cells in Pompe disease remain functional and responsive to at least some upstream signals; given the undiminished satellite cell reserve found in Pompe patient muscle biopsies, these findings suggest that identifying and then correcting and/or bypassing the failed step in satellite cell activation may lead to a new and more effective therapy for the devastating skeletal myopathy in Pompe disease. One potential avenue for additional investigation, suggested by both groups, is to investigate whether exercise (which was shown to activate satellite cells in healthy human volunteers [12]) will be able to induce satellite cell activation in the mouse models of Pompe disease. Another approach will be to elucidate the molecular and cellular basis that underlies ineffective satellite cell activation in Pompe skeletal muscle. Given that the bioenergetic demands of activated satellite cells are met through autophagy induction [1, 13] and that autophagy impairment contributes to pathogenesis of Pompe disease [6], both groups raised the possibility that deficiency in autophagy may provide an explanation for the observed failure of satellite cell activation in this disorder. If this mechanism turns out to be correct, the findings will have implications beyond Pompe disease, with benefits potentially extending to patients with other skeletal myopathies characterized by impaired autophagy (such as Dannon disease, X-linked myopathy with excessive autophagy, and inclusion body myositis).
